# Lack of CT scanner in a rural emergency department increases inter-facility transfers: a pilot study

**DOI:** 10.1186/s13104-017-3071-1

**Published:** 2017-12-28

**Authors:** Catherine Bergeron, Richard Fleet, Fatoumata Korika Tounkara, Isabelle Lavallée-Bourget, Catherine Turgeon-Pelchat

**Affiliations:** 1 Chaire de recherche en médecine d’urgence de l’Université Laval, CHAU Hôtel-Dieu de Lévis, 143 Rue Wolfe, Lévis, QC G6V 3Z1 Canada; 2 Department of Family Medicine and Emergency Medicine, Université Laval, 1050, Avenue de la Médecine, Québec City, QC G1V 0A6 Canada

**Keywords:** Emergency department, Rural, Computed tomography, Inter-facility transfer

## Abstract

**Objective:**

Rural emergency departments (EDs) are an important gateway to care for the 20% of Canadians who reside in rural areas. Less than 15% of Canadian rural EDs have access to a computed tomography (CT) scanner. We hypothesized that a significant proportion of inter-facility transfers from rural hospitals without CT scanners are for CT imaging. Our objective was to assess inter-facility transfers for CT imaging in a rural ED without a CT scanner.

**Results:**

We selected a rural ED that offers 24/7 medical care with admission beds but no CT scanner. Descriptive statistics were collected from 2010 to 2015 on total ED visits and inter-facility transfers. Data was accessible through hospital and government databases. Between 2010 and 2014, there were respectively 13,531, 13,524, 13,827, 12,883, and 12,942 ED visits, with an average of 444 inter-facility transfers. An average of 33% (148/444) of inter-facility transfers were to a rural referral centre with a CT scan, with 84% being for CT scan. Inter-facility transfers incur costs and potential delays in patient diagnosis and management, yet current databases could not capture transfer times. Acquiring a CT scan may represent a reasonable opportunity for the selected rural hospital considering the number of required transfers.

## Introduction

Quebec is Canada’s second largest province, with 20% of its population living in rural areas [1, 2]. Rural patients cope with a curtailed physician-population ratio [3], longer pre-hospital emergency care response times [4] and greater travel distances [5, 6] than urban patients. Rural patients and practitioners also live and work in the context of limited access to specialists [6–9] and resources including, diagnostic imaging tools [6–15].

Computed tomography (CT) scans are commonly used for the diagnosis of many surgical and time-sensitive emergency conditions such as stroke, head trauma and pulmonary embolism [16]. Most urban emergency departments (EDs) in Canada have access to 24/7 in-hospital CT scanners, along with more than 90% of all US hospitals [17]. The use of CT scans in EDs has increased 330% between 1996 and 2007, and approximately 25% of all CT scans performed in the US are now requested by the ED [18].

In Canada, rural EDs outside the province of Quebec have poor access to CT scans; 15% of Canadian rural EDs have access to a CT scanner, compared to 77% in Quebec [6–8]. This finding has generated debate on a national scale [19]. While scanners may sometimes be overused, lack of a local CT scanner may impose considerable burden on the physician decision-making process in rural settings where inter-facility transfers must to be weighed in regards to the risks of transport over great distances, delayed diagnosis, treatment and costs.

In a Canadian study conducted 18 years ago, up to 14% of inter-facility transfers from five rural hospitals to referral centres were solely for CT scans [9]. Another report suggested that rural EDs are responsible for up to 60% of patient transfers to tertiary centres, transfers that could have been avoided if the rural EDs had radiology services [14]. Similarly, an American group reported that patients undergoing CT scans at the hospital to which they were initially admitted were less likely to be transferred [20].

Although Canada’s Health Act has a clause promising the right to access health care [21], there is no specific guideline concerning standards for access to CT scans [22, 23]. According to a thorough review of the literature, few recent studies have examined inter-facility transfers for CT scans (“Annex 1”), particularly in rural Canada, where CT imaging is limited. This pilot project aimed to assess inter-facility transfer requirements for CT imaging in a rural ED without local access to a CT scanner.

## Main text

### Methods

This pilot project is derived from a previous study [6, 24]. The original study protocol was approved by the CSSS Alphonse–Desjardins Research Ethics Committee (Project MP-HDL-1213-011). In this earlier study, we collected data on all of Quebec’s rural EDs (N = 26). We found that only 6 out of 26 (23%) rural EDs did not have access to a 24/7 CT scanner. For the purposes of the current pilot study, we selected one of these six rural EDs for convenience reasons (relative proximity to research team and previous enthusiastic participation in pilot stages of studies). We henceforth refer to this hospital as the “selected rural ED”.

We contacted the selected rural hospital’s medical archivists to obtain data on ED visits and transfer details from 1 April 2010 to 31 March 2015. Using the local ED triage software program, *StatUrgence,* we collected the following data: total number of ED visits, total number of patient transfers, receiving hospital names, and their distances from the selected rural ED (“Annex 2”). Distances between hospitals were measured using Google Maps [25, 26].

Additional data was collected concerning inter-facility transfers from the selected rural ED to another rural referral hospital. This hospital, 50.9 km distant, was similar to the selected rural ED except that it had a CT scanner. Transfers to this facility were therefore likely for the purpose of a CT scan, while transfers to the more distant urban, academic hospitals were more likely for severe cases requiring specialized imaging and consultants. We henceforth refer to this rural hospital as the “rural referral centre”. We collected data on transfers between these two rural hospitals between 1 April 2010 and 31 March 2015. Only transfer requests from the ED were considered; thus, CT imaging requests from admission beds, local clinics, family doctors or specialists outside the ED were excluded. Both urgent and elective transfers were eligible, regardless of the means of transportation. The local archivist compiled the electronic medical records for all patients transferred to the rural referral centre during our study period in *StatUrgence* which provided the date of transfer and the escort needed for each transfer.

Two medical students (CB, IL-B.) independently reviewed transfer patients’ corresponding medical records in *MédiRad*, the rural referral centre’s radiology software, to determine which patients were transferred for a CT scan. They searched the software to verify whether patients underwent CT scan imaging on their transfer date, and if so, double-checked if the origin of the scan request corresponded with the selected rural ED. If it did not, it was concluded that the transfer was not for a CT scan. For patients transferred for a CT scan, we searched for four main variables in *MédiRad*: age, sex, type of scan, and the interval between the request and the scan. To calculate the interval, we subtracted the time the scan was ordered in the ED from the time the scan was conducted as noted in the radiologist’s reports. We only calculated the delay for urgent scan requests in the selected ED; we excluded elective scans.

The primary outcome of our study was the preliminary results of the inter-facility transfers for a CT scan in a Quebec rural ED without a CT scanner to another similar rural hospital with a scanner.

### Results

#### Inter-facility transfers for a CT scan

Characteristics of the population served by the selected ED and facilities available to them are described in Table 1.

**Table 1:** Sociodemographic characteristics of the selected rural hospital in 2011

Local population	7332
Population density per square kilometer	13.4
Median age of the population	51 years
Distance to nearest trauma center	91.8 km
Hospital services	Laboratory, X-ray, portable ultrasound

Data from statistics Canada [1]

Over a 5-year period, the selected ED received an average of 13,341 ED consultations per year, 444 (3%) of which were transferred to other facilities. One-third of these transfers (148, or 33.2%) were to the rural referral centre. Of patients transferred to this referral centre, 125 (84%) were transferred to perform a CT scan, i.e. 28% (n = 125/444) of all transfers from the selected ED (Table 2). Of these 125 transfers, 3.4% required a nurse escort. Finally, as a yearly average, 330 (74%) of the transfers from the selected rural ED were by ambulance, with approximately 93 (63%) transfers to the rural referral hospital for a CT scan.

**Table 2:** Characteristics of selected rural ED: visits and transfers

	2010	2011	2012	2013	2014	AVERAGE
Selected ED visits	13,531	13,524	13,827	12,883	12,942	13,341
Selected ED total patient transfers	456	433	395	448	488	444
	3.4%	3.2%	2.9%	3.5%	3.8%	3.3%
Selected ED patient transfers to rural referral hospital	131	145	129	156	177	148
	28.7%	33.5%	32.7%	34.8%	36.3%	33.2%
Selected ED transfers to rural referral hospital for a CT scan	101	116	108	140	158	125
	77.1%	80.0%	83.7%	89.7%	89.3%	84.0%

### Discussion

#### Inter-facility transfers for CT scans

The selected ED transferred 3.3% of its patients, a higher percentage than the approximately 2% cited in literature [9, 27, 28]. One third of all transfers went to the rural referral centre, and 84% of these transfers were in fact for CT scans. Thus, at least 28% of all the ED’s inter-facility transfers were exclusively required for CT imaging, which is twice as much as the proportion reported in the literature [9]. Since this proportion is based on transfers to only one rural referral centre (that is the “designated” CT imaging center for the region) and only from the ED (not from admitted patients or local clinics) this may be an underestimation of overall transfers for CT scans.

Inter-facility transfers are costly and can delay the diagnosis and management of time-sensitive emergency conditions. The selected rural ED is 50.9 km away from the rural referral hospital, and in the opposite direction of the nearest Level 1 trauma center (91.8 km away). Travel is accomplished in mountainous country road conditions that are often hazardous, particularly in winter. In this region, a single ambulance transfer takes 3 h round trip and costs $722 including paramedic care [29–31]. Thus our rural ED’s estimated average of 93 ambulance transfers for a CT scan cost the healthcare system approximately $68,000 per year, not including healthcare professionals’ or staff time during transfer or direct and indirect costs accrued by the patient. Inter-facility transport costs for CT imaging must be weighed against the costs necessary to purchase and maintain a local scanner (upwards of $730,000 for purchase and $160,000/year maintenance [32]).

In addition to reducing inter-facility transports, it has been shown that rural CT scans both narrow the gap between urban and rural levels of health care as well as promote general patient and local care because of the faster access to diagnoses, higher confidence in diagnoses, quicker treatments, better management of referrals to specialists, and lower waiting times for CT scans for rural patients [12]. Walkerton is a good example of the beneficial impacts of access to a CT scanner in a rural setting: this pilot project had such conclusive results that the study was ended early and Walkerton decided to keep the scanner [12].

#### Feasibility

This pilot study also indicates that conducting a larger study is meaningful and feasible. Data on inter-facility transfer requirements for CT imaging is important and this was easily and reliably obtained using current databases. We had access to all essential transfer information, except for the time intervals between the CT requests and the actual CT imaging. Only 2.5% of all patient records mentioned the time at which the scan was ordered in the ED, so we could not expect significant findings on delays. This hinders our capacity to estimate potential delays in diagnosis and treatment. Quebec’s rural hospitals have limited electronic databases [29]. Inter-facility data and imaging time-frames are critical for resource planning and should be included in future iterations of electronic databases. Without these changes, only prospective and more costly study where each inter-facility transfer for CT is tracked from time requested to image interpretation and physician assessment would help us assess the impact of not having access to a local CT scanner.

#### Strengths

- We believe this is the first Canadian study in the last 15 years to evaluate inter-facility transfers from a rural ED without a CT scan [9].
- Considering our findings that less than 15% of rural EDs in Canada have no in-hospital access to CT scans, and faced with great transfer costs, similar provincewide or nationwide studies are warranted [7, 31].

### Conclusion

A considerable proportion of inter-facility transfers were required for CT imaging in a small rural hospital ED. Inter-facility transfers incur costs and potential delays in patient diagnosis and management, yet current databases could not capture transfer times and final diagnoses. Further improvement of databases is required. Finally, acquiring a CT scan may represent a reasonable solution for the selected rural hospital considering the number of required transfers. Other studies are justified to help stakeholders decide on the purchase of a CT in rural hospitals.

## Limitations

- This pilot study was conducted in a single site out of the 6/26 potential rural hospitals in Quebec without access to a CT scanner. Our findings may not be generalizable to these 5 other EDs, where ED volumes and distances to CT may be different.
- The retrospective design may have limited thorough data review in charts. For example, we did not have access to data concerning reasons for transfer nor the time intervals from requests and the imaging interpretation. Moreover, the retrospective nature of the data makes it impossible to prove that the patients were solely transferred for a CT scan; it is plausible that some patients may have been transferred for another reason than for a CT, and received a scan afterward in the referral hospital. Whatsoever, if the patients had not been transferred, they could not have received this particular investigation in the selected rural ED.

## Figures and Tables

**Fig. 1:**
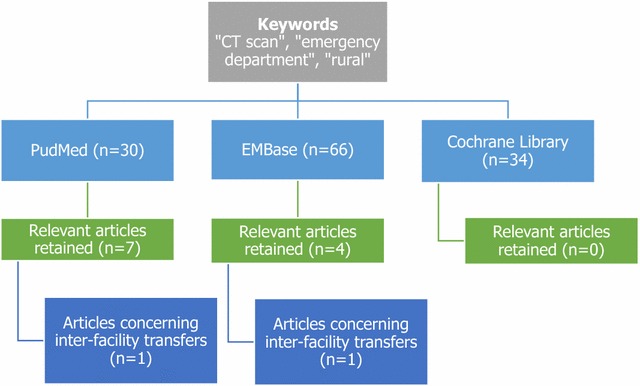
Review of the literature, July 2015

## Annex 1

See Fig. 1.

All articles were reviewed on the basis of the title and abstract. We retained 11 relevant articles. We defined as relevant articles that were about emergency rural CT scans: accessibility, inter-facility transfers, quality of care, difference with urban trends, etc. There were no restrictions on origin or language of publication. However, we rejected articles about telemedicine in rural areas, rural stroke systems and very specific diseases. We also discarded articles more than 20 years old since CT scan use in rural areas evolved considerably over this period. Of the 11 relevant articles, only two concerned inter-facility transfers.

### Search strategies

#### PubMed

“Tomography, X-Ray Computed”[Mesh] OR “CT”[tiab] OR “scan”[tiab] OR “CT scanners”[tiab] OR “Computed Tomography”[tiab]) OR “CT scanner”[tiab] OR “CTscan”[tiab] OR “CT scanning”[tiab].

“Emergency services”[TIAB] OR “emergency service” [TIAB] OR “emergency departments” [TIAB] OR “emergency department”[TIAB] OR “emergency medical services”[mesh] OR “emergency medical service” [tiab] OR “Emergency Service, Hospital”[mesh].

“Rural health services” [mesh] OR “rural health service” [tiab] OR rural population [mesh] OR “remote area” [TIAB] OR “remote areas”[TIAB] OR “rural healthcare” [TIAB] OR “Rural Health”[mesh] OR “medically underserved area” [mesh] OR “medically underserved areas” [tiab] OR “rural emergency department”[tiab] OR “rural emergency departments”[tiab] OR “rural emergency care”[TIAB].

#### EMBase

‘computed tomography scanner’/exp OR ‘CT’:ti,ab OR ‘scan’:ti,ab OR ‘CT scanners’:ti,ab OR ‘Computed Tomography’:ti,ab OR ‘CT scanner’:ti,ab OR ‘CTscan’:ti,ab OR ‘CT scanning’:ti,ab.

‘emergency medical services education’/exp OR ‘emergency health service’/exp OR ‘emergency services’:ti,ab OR ‘emergency service’:ti,ab OR ‘emergency departments’:ti,ab OR ‘emergency department’:ti,ab OR ‘emergency medical service’:ti,ab.

‘rural health care’/exp OR ‘rural population’/exp OR ‘health care planning’/exp OR ‘rural health service’:ti,ab OR ‘remote area’:ti,ab OR ‘remote areas’:ti,ab OR ‘rural healthcare’:ti,ab OR ‘medically underserved areas’:ti,ab OR ‘rural emergency department’:ti,ab OR ‘rural emergency departments’:ti,ab OR ‘rural emergency care’:ti,ab.

#### Cochrane library

(CT OR scan OR CT scanners OR Computed Tomography OR CT scanner OR CTscan OR CT scanning):ti,ab,tb OR (Tomography, X-Ray Computed):kw.

(Emergency services OR emergency service OR emergency departments OR emergency department OR emergency medical service):ti,ab,tb OR (emergency medical services OR Emergency Service, Hospital):kw.

(Rural health service OR remote area OR remote areas OR rural healthcare OR medically underserved areas OR rural emergency department OR rural emergency departments OR rural emergency care):ti,ab,tb OR (Rural health services OR rural population OR Rural Health OR medically underserved area):kw.

## Annex 2

See Table 3.

**Table 3:** ED transfers from selected rural hospital to all referral centres, with distances

Years	Total ED patient transfers	Referral centres (and distances)		
		Primary care rural hospital with CT scan (50.9 km)	Urban teaching hospital (91.8 km)	Other
2010	456	131	310	15
2011	433	145	283	5
2012	395	129	256	10
2013	448	156	283	9
2014	488	177	303	8
Total average	444	147.6	287	9.4
Percentage (%)	–	33.2	64.6	2.1

## Abbreviations

EDs: rural emergency departments
CT: computed tomography
